# Neural acetylcholinesterase and monoamine oxidase deregulation during streptozotocin-induced behavioral, metabolic and redox modification in *Nauphoeta cinerea*

**DOI:** 10.1186/s12868-024-00890-z

**Published:** 2024-08-29

**Authors:** Opeyemi B. Ogunsuyi, Olawande C. Olagoke, Mayokun E. Famutimi, Damilola M. Olatunde, Diogo O. G. Souza, Ganiyu Oboh, Nilda V. Barbosa, João B.T. Rocha

**Affiliations:** 1 Departamento de Bioquímica e Biologia Molecular, Programa de Pos-graduacao em Bioquimica Toxicologica, Centro de Ciências Naturais e Exatas (CCNE), Universidade Federal de Santa Maria, Santa Maria, RS 97105-900 Brazil; 2 Department of Biomedical Technology, Federal University of Technology, P.M.B. 704, Akure, Nigeria; 3 Drosophila Research Lab, Functional Foods and Nutraceuticals Unit, Federal University of Technology, P.M.B. 704, Akure, Nigeria; 4 Department of Medicine, Division of Gastroenterology, Beth Israel Deaconess Medical Center, Harvard Medical School, Boston, MA USA; 5 Department of Medicine, Division of Translational Research and Technology Innovation, Beth Israel Deaconess Medical Center, Harvard Medical School, Boston, MA USA; 6 Department of Physiology, Kampala International University, Ishaka-Bushenyi, Uganda; 7 Department of Biochemistry, Federal University of Technology, P.M.B. 704, Akure, Nigeria; 8 Departamento de Bioquímica, Instituto de Ciências Básicas da Saúde, Universidade Federal do Rio Grande do Sul, Rua Ramiro Barcelos 2600-Anexo, Porto Alegre, RS 90035-003 Brazil

**Keywords:** 3Rs, Redox-inflammation crosstalk, Neurodegeneration, Oxidative damage, Neurotoxicity

## Abstract

Genetic and environmental factors have been linked with neurodegeneration, especially in the elderly. Yet, efforts to impede neurodegenerative processes have at best addressed symptoms instead of underlying pathologies. The gap in the understanding of neuro-behavioral plasticity is consistent from insects to mammals, and cockroaches have been proven to be effective models for studying the toxicity mechanisms of various chemicals. We therefore used head injection of 74 and 740 nmol STZ in *Nauphoeta cinerea* to elucidate the mechanisms of chemical-induced neurotoxicity, as STZ is known to cross the blood-brain barrier. Neurolocomotor assessment was carried out in a new environment, while head homogenate was used to estimate metabolic, neurotransmitter and redox activities, followed by RT-qPCR validation of relevant cellular signaling. STZ treatment reduced the distance and maximum speed travelled by cockroaches, and increased glucose levels while reducing triglyceride levels in neural tissues. The activity of neurotransmitter regulators – AChE and MAO was exacerbated, with concurrent upregulation of glucose sensing and signaling, and increased mRNA levels of redox regulators and inflammation-related genes. Consequently, STZ neurotoxicity is conserved in insects, with possible implications for using *N. cinerea* to target the multi-faceted mechanisms of neurodegeneration and test potential anti-neurodegenerative agents.

## Introduction

Advances in medicine and technology have increased the average life expectancy, especially in the global north, but ageing is strongly linked with neurological disorders that affect the brain and some other regions of the nervous system, including neurodegenerative diseases that are often irreversible, progressive, and characterized by diminished cognitive abilities, muscle weakness and locomotor difficulties [1]. Regardless of the cause, neurodegenerative diseases often portray features of neuronal loss or damage, and the type of disorder depends on the extent and site of damage [2]. However, the heterogeneous nature of population-based genetics and the complexities in inheritance patterns increase the difficulty of pathway analysis of human data, making it important to exploit models that may explain the pathophysiological mechanisms driving changes in protein structure and function across multiple generations [3, 4].

Insect models like *Drosophila melanogaster* and *Nauphoeta cinerea* have been insightful in depicting fundamental propositions like the chromosomal theory of inheritance or mechanistic patterns like xenobiotic-related neurotoxicity [5–7]. The lobster cockroach has a high fecundity rate, with each female producing about 20 oothecas that house 26 to 40 eggs [8], it is cheap and easy to maintain in a laboratory setting, and the neural tissues are more accessible given the comparative larger size to the Drosophila. Importantly, although the insect open circulatory system is different from the mammalian closed circulatory system, the brain in insects and mammals is protected from fluctuations in solute concentration by the hemolymph-brain barrier and blood-brain barrier respectively [9], and metabolic pathways are conserved from insects to mammals [10–12]. For example, ablation of insect insulin-producing cells (IPC) creates similar phenotypes to those seen in mammals with beta cell damage, even though insect IPCs are found in the brain and mammalian beta cells are found in the pancreas [13].

The tropism and toxicity of streptozotocin (STZ) towards the mammalian beta cells induces DNA alkylation [14] and nicotinamide adenine dinucleotide depletion [15] with a consequent disruption in glucose energy metabolism. Likewise, intracerebroventricular (ICV) administration of STZ in rodents induces insulin resistance in the brain that results in hyperphosphorylation of tau proteins and aggregation of Aβ in meningeal vessels, which causes neural and behavioral changes that are reminiscent of sporadic Alzheimer disease [16]. Moreover, STZ is mutagenic to the mosquito cell [17], and the intraabdominal administration of STZ in the lobster cockroach alters brain glucose metabolism, including upregulation of glucose transporter expression, increased glucose absorption into neural tissues and deregulation of redox balance [12, 18, 19]. Similar phenotypes are therefore seen across insects and rodents that are exposed to STZ.

Nonetheless, several knowledge gaps remain in the understanding of the neurodegenerative process, which may explain why current management plans are heavily focused on alleviating symptoms, instead of providing cures. For example, the limited understanding of neurobehavioral plasticity persists from insects to mammals. Given the homology between insect and mammalian signaling [12, 20], and the reports of the effectiveness of using cockroaches to illustrate the interaction of chemicals with living systems [21–25], we used *Nauphoeta cinerea* to explore chemical-induced neurotoxicity, to provide insights into the mechanisms driving neurodegeneration, while advancing the prospect of replacing, reducing and refining (3Rs) animal use in biomedical research.

## Materials and methods

### Reagents

Streptozotocin (Sc-200719 A) was sourced from Santa Cruz Biotechnologies, Germany. Chemical reagents such as acetylthiocholine iodide, reduced glutathione, and ferrous sulphate, were procured from Sigma Aldrich Co. (St Louis, Missouri, USA). Trichloroacetic acid (TCA) and sodium acetate were sourced from Sigma Aldrich (Steinheim, Germany). Acetic acid, hydrochloric acid, aluminum chloride, and potassium acetate, were obtained from BDH Chemicals Ltd., (Poole, England). Except stated otherwise, all other chemicals and reagents were of analytical grades and the water was glass distilled.

### *N. Cinerea* stock culture

*N. cinerea* was raised at the Biochemistry Department from UFSM, RS, Brazil. The cockroaches were maintained and reared on a commercial dog chow at constant temperature at constant temperature (24 ± 3 °C) and humidity (57–75%).

### Experimental design

40 size-matched cockroaches (including male and female) were randomly selected per study group and were housed in plastic containers. The inner edges of the housing containers were lubricated to prevent the cockroaches from crawling out, after which the cockroaches were weighed (mg) using a weighing scale. There were three groups in total: the control group, the 74 nm STZ injected group, and the 740 nm STZ injected group. A preliminary study was done to test a sham injection of 0.8% NaCl, but there was no difference in the biochemical properties of untreated and sham-injected cockroaches, hence untreated cockroaches were used as the control group. Streptozotocin concentrations were based on previously reported pilot studies in *Nauphoeta cinerea* that were extrapolated from data on the injection of *β-*cell cytotoxic agents in moths and rodents [18, 26, 27].

All groups were anaesthetized using ice. The period of anaesthesia was about 5 min to reduce the agility of the cockroaches during induction. Care was taken not to keep the cockroaches under anaesthesia for longer than 5 min to prevent death before induction. Single-use insulin syringes were then used to administer 20 µL STZ (74 nm and 740 nm respectively) directly to the central axes of the cockroach heads, and the gross toxicity of STZ was determined in terms of insect survival [18]. 1.7 g of flour, 0.2 g of sugar, 0.05 g of casein, 0.01 g of salt and 0.04 g of milk were weighed using a High Precision Balance and were thoroughly but carefully mixed using a Vortex mixer [18]. The cockroaches had access to water and feed *ad libitum* and were observed for 7 days, after which behavioural profiles were assessed.

### Neurolocomotor assessment

The cockroaches were carefully transferred into a white plastic box (a new environment) 19 cm in length, 12.5 cm in width and 5 cm in height and their behavior was filmed during a 10 min trial period using an overhead-mounted webcam. Behavioural endpoints of locomotor activity such as total distance travelled, maximum speed, total time immobile, and total time in periphery were analyzed from the video files using the video-tracking software; ANY-maze 6.0, Steolting, CO, USA, as earlier described [28, 29].

### Biochemical analysis

The cockroach heads were excised using surgical blades. Three heads per vial (*n* = 5) were then weighed, homogenized in 0.1 M phosphate buffer, pH 7.4, and centrifuged at 2500 g for 10 min at 4 ^o^C. The resulting tissue was used to carry out biochemical assays. Total protein was estimated by applying 2 µL tissue homogenate to the sensor of a NanoDrop 2000 spectrophotometer (ThermoFisher Scientific Inc, USA) and reading the absorbance at 280 nm as earlier described [30, 31].

#### Head glucose and triglyceride content

Glucose content was determined using a glucose oxidase kit (Lot: 07135A0217) according to the manufacturer’s protocol (Quimiglic-Ox, Brazil). In brief, 196 µL glucose reagent and 4 µL tissue was added to the sample wells, while the blank well was made up of 196 µL glucose reagent and 4 µL distilled water. The standard well was made up of 4 µL glucose standard and 196 µL glucose reagent. The mixture was then incubated for 10 min at 37 ^o^C, after which it was read at 505 nm in a BIO-RAD Microplate reader.

Triglyceride content was determined using a triglyceride kit according to the manufacturer’s protocol (Biotecnica, Brazil) In brief, 196 µL triglyceride reagent and 4 µL tissue was added to the sample wells, while the blank well was made up of 196 µL triglyceride reagent and 4 µL distilled water. The standard well was made up of 4 µL triglyceride standard and 196 µL triglyceride reagent. The mixture was then incubated for 10 min at 37 ^o^C, after which it was read at 505 nm in a BIO-RAD Microplate reader [18].

#### Acetylcholinesterase and monoamine oxidase activity

Acetylcholinesterase activity was carried out in an assay medium consisting of (at final concentration) 10 mM phosphate buffer (pH 7.4), 1 mM 5,5-dithio-bis (2-nitrobenzoic) acid (DTNB), and 0.8 mM acetylthiocholine iodide, 30 µL of tissue sample and the total reaction volume made up to 200 µL with distilled water. The mixture was then read using a SpectraMax Microplate Spectrophotometer at 412 nm (15s intervals for 30 min). The AChE activity was thereafter calculated and expressed as mmolAcSch/h/mg protein [21].

Monoamine oxidase (MAO) activity was determined as previously reported [32, 33]. The reaction consisted (final concentration) of 72 mM potassium phosphate buffer (pH 7.4), 0. 5 mM benzylamine, 50 µl of tissue homogenate, and 50 µl of distilled water. This was followed by incubating the mixture for 30 min at 25 degrees Celsius and adding 300 µl of 10% perchloric acid (5.2% of total reaction volume). The mixture was thereafter centrifuged at 1,500 g for 10 min. The MAO activity was monitored at 280 nm and expressed as mmol/mg protein.

#### Oxidative stress assay

Total reactive oxygen and nitrogen species were assayed in a reaction which included 5 µM 2′, 7′-dichlorofluorescein diacetate (2, 7-DCFDA), 5 µL of homogenate, and 75 mM potassium phosphate buffer (pH 7.4). After that, for 30 min at 30-second intervals, the fluorescence emission was observed using a Spectra Max spectrofluorometer (excitation = 480 nm; emission = 525 nm). The results were represented as a change in fluorescence intensity per minute. [21, 23, 34].

The reactive oxygen species (ROS) level in the tissue homogenates was also estimated as H_2_O_2_ equivalent according to the method of Hayashi et al. [35] as previously reported by Oboh et al. [36]. In brief, 5 µl of tissue homogenate and 57 mM sodium acetate buffer (pH 4.8) were incubated for 5 min at 37 degrees Celsius. Thereafter, 2.5 mg/mL of n-n-diethyl-para-phenylenediamine (DEPPD) and 1.8 µM of ferrous sulphate solution were added to the mixture. The absorbance was measured at 505 nm using a spectrophotometer. ROS levels was estimated from an H_2_O_2_ standard calibration curve and expressed as Unit/mg protein, where 1 unit = 1 mg H_2_0_2_/L.

The lipid peroxidation was estimated using a mixture of 50 µL tissue, 150 µL 8.1% SDS, 250 µL 20% acetic acid in hydrogen chloride (p.H 3.4) and 250 µL 0.6% TBA for the sample test tubes; and 50 µL distilled water, 150 µL 8.1% SDS, 250 µL 20% acetic acid in hydrogen chloride (p.H 3.4) and 250 µL 0.6% TBA in the blank test tube. The mixture was then incubated for 1 h at 95 ^o^C, after which it was read at 532 nm in a visible Spectrophotometer [37, 38].

#### Antioxidant and detoxification activity

Using Ellman et al.‘s method [39], the amount of total thiol content in the tissue homogenate was measured. The reaction mixture included 20 µL of tissue homogenate, 0.5 mM 5,5’-dithiobis-(2-nitrobenzoic acid) (DTNB), and 85 mM potassium phosphate buffer (pH 7.4). The total reaction volume was 200 µL. The same quantity of tissue was added to reaction blanks, but DTNB was not present. After that, the sample was allowed to incubate for 30 min at room temperature, and the absorbance was measured at 412 nm. After that, the total thiol content was determined and represented as (mmol/mg protein).

Glutathione-S-transferase activity was assayed according to the method of Habig and Jakoby with slight modifications, using 1-chloro-2,4-dinitrobenzene (CDNB) as a substrate [40, 41]. This assay consists of 100 µL of tissue homogenate, 1. mM ethylenediaminetetraacetic acid, 0.80 mM chloro-2, 4-dinitrobenzene (CDNB), 3.20 mM glutathione as substrate and 70 mM potassium phosphate buffer (pH 7.0). After ten minutes, the GST activity was measured using a spectrophotometer (Spectra Max) plate reader at 340 nm. The result was expressed as unit/mg protein where 1 unit = µmol/ml/min/mg protein.

### Real-time polymerase chain reaction

Our *Nauphoeta cinerea* transcriptome [12, 42] was queried with the existing insect gene sequences and the ensuing Nauphoeta gene sequences were used to design primers as earlier described [18, 19] and shown in Table 1. A reverse transcription-quantitative real-time polymerase chain reaction was used to evaluate the mRNA expressions. Fastzol™ reagent (Lot: 211711 by Quatro G Biotecnologia, Brazil) was used to extract the total RNA from the cockroach head. The concentration of RNA in the samples was measured with a Nanodrop400A™, thereafter, it was visualized in a 1.5% agarose gel ad then treated with DNase I (M0303S by New England Biolabs). The High-capacity cDNA Reverse Transcription Kit (Lot: 4368814 by Applied Biosystems) was used to synthesize 1 µg of cDNA in a thermal cycler (BIO-RAD, USA). All levels of expression were normalized to TBP. Real-time PCR was carried out using a QuantStudio 3 RT-qPCR System (ThermoFisher Scientific, USA). Each well contained a final volume of 20 µL, 2.5 ng/µL cDNA, 10 µL MasterMix PCR Tempo Real - SYBR Green/ROX (Lot: P242401001 by Quatro G Biotecnologia, Brazil) and 0.25 µM of forward and reverse primers (Table 1). The PCR entailed 40 thermal cycles of 15 s at 94 °C, 10 s at 60 °C, and 30 s at 72 °C, and a melt curve stage with 1 cycle of 94 °C for 10 s, 55 °C for 1 min and 94 °C for 15 s. Primer efficiency was determined using a five-point dilution of a pool of samples [19], and the SYBR fluorescence was measured using the StepOne™ design and analysis program [43]. The experiments were performed in triplicates, and the 2^−ΔΔCT^ approach [44] was applied to calculate gene expression levels.

**Table 1:** Primer sequence for RT-qPCR assay

Genes	Primer sequence	Ref ID*
*GLUT1*	F – AAATATGGACACCGCGAGAG. R – TGCCCCGGAAGTGAATATAG	9E-2023-03-2836
*PI3K*	F – TTGCCGACAGACATTCAGAC R – AATGGGACACGTTCTCTTCG	9E-2023-03-2837
*DUOX*	F – TGGGCCTTGGACTACAAAC. R – AAATTGATACCAGTCCTGATGC	9E-2023-03-2817
*GST theta*	F – AAGAAATGGGTTGCAGGAGA. R – CATTGGGATGCTTGCTTACA	9E-2023-03-2804
*SOD*	F – GTATTCTGGTGGCTGCGAAA. R – TAAACCCAACACAGAGCCTTG	9E-2023-03-2813
*Catalase*	F – ACGAGATCCAGCATCTGACC. R – CTCCACGGTTATCCACAGGT	9E-2023-03-2812
*EGR*	F – CACTTGTATGGCAGGTATTGGA. R – GGATGAACACGATGTAAATGGA	9E-2023-03-2832
*TOLL1*	F – TTGTGTTTCTGGACATCAGTCATAA. R – CATGCAGATTGTTGTGTTCCA	9E-2023-03-2808
*UPD3*	F – GGAACATCCACCTCTGATCG. R – TAGGAGGACCCGAGAATGTG	9E-2023-03-2833
*TBP*	F – GGTGCGAATGTGGAGTACAG. R – TAGTGGCTCCAGTGCAAGTC	9E-2023-03-2839

* Reference ID for each primer, assigned by the manufacturer – Exxtend Solução, Brasil

### Statistical analysis

The results were expressed as mean ± standard deviation (SD) and were analyzed by one-way Analysis of Variance (ANOVA) after the Shapiro-Wilk Test for normality. Post hoc analysis was carried out with Tukey’s multiple comparisons test. All statistical analysis was carried out using the software Graph pad PRISM (V.8.0), and significance was set at *p* ≤ 0.05.

## Results

### Survival and behavioural monitoring

**Fig. 1:**
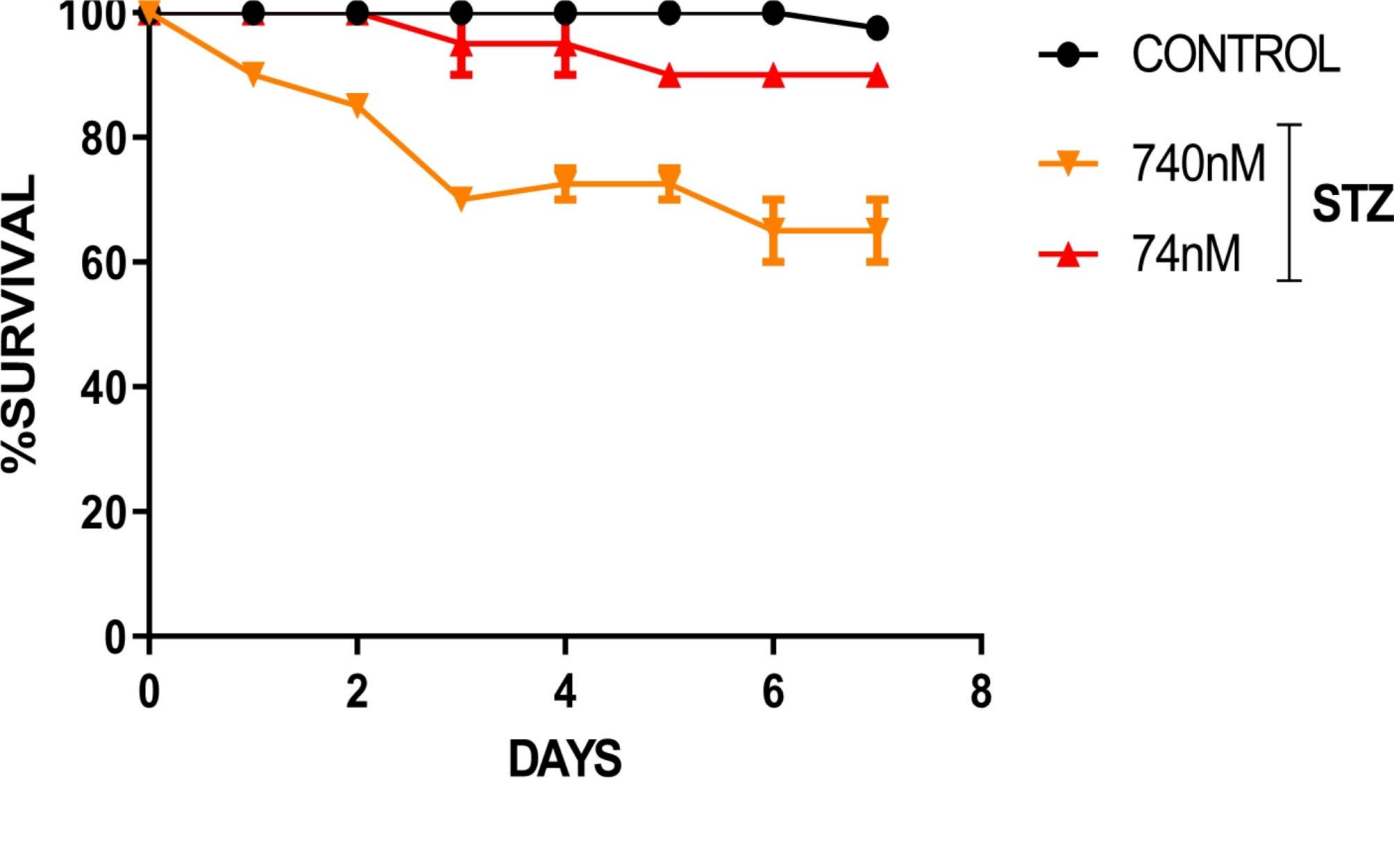
7-day Kaplan-Meier survival analysis of cockroaches after single dose head STZ injection (*N* = 40). Log-rank (Mantel-Cox) test showed significant (*P* = 0.0003) reduction in survival (increased mortality) of 74 and 740 nM STZ-treated cockroaches compared with control

**Fig. 2:**
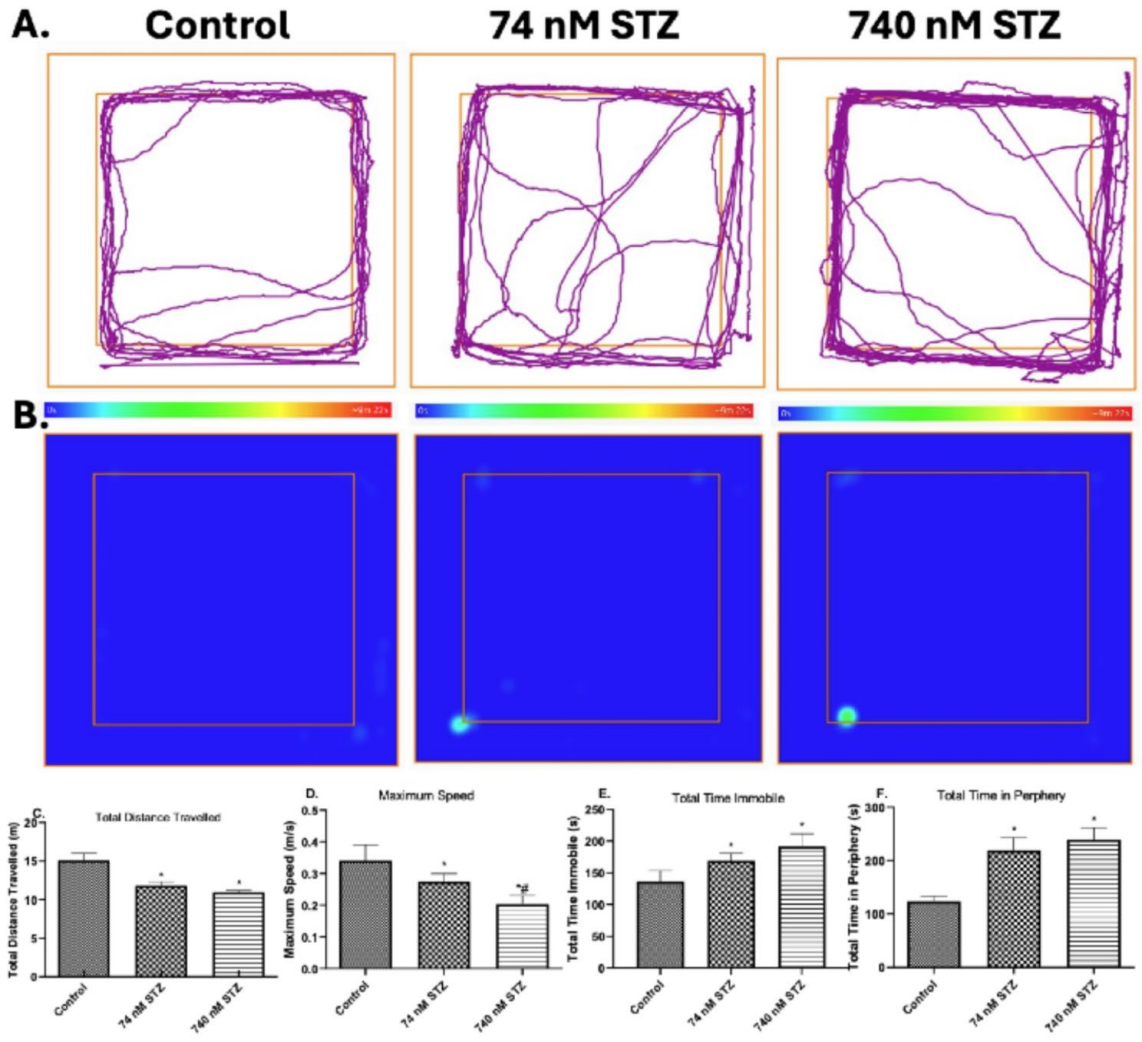
Motor and exploratory profile of cockroaches, 7 days after head STZ injection. *n* = 8. 8-minute video recordings of (**A**) track plot and (**B**) heat map were analysed with the ANY-maze (Stoelting CO, USA) software. One-way ANOVA with Tukey’s multiple comparisons test indicated a significant decrease in (**C**) total distance travelled and (**D**) maximum speed, as well as a significant increase in (**E**) total time immobile and (**F**) total time in periphery in cockroaches exposed to 74 and 740 nmol STZ head injection, compared with cockroaches in the control group. All values are mean ± SD. * indicates a significant difference from control

We recorded the trends in mortality rate, as well as behavioural responses to 74 and 740 nmol head STZ injection and found significantly diminished survival in the treated group compared with the control group. Expectedly, there was higher mortality in the 740 nmol STZ treated group compared with the 74 nmol STZ treated group (Fig. 1). Head STZ injection also disrupted *N. Cinerea* motor and exploratory activities (Fig. 1A **& B**), as there was a significant reduction in the total distance travelled [F (2,21), = 12.66; *P* = 0.0002; Fig. 2C] and maximum speed [F (2,21), = 29.61; *P* < 0.0001; Fig. 2D] of the treated groups in relation to control. Conversely, the treated group spent significantly more time immobile [F (2,16), = 18.3; *P* < 0.0001; Fig. 2E] and in the periphery [F (2,17), = 6.9; *P* < 0.005; Fig. 2F] compared with the control group. However, the behavioral endpoints were not significantly different across the treatment (74 and 740 nmol STZ) groups.

### Biochemical analyses

**Fig. 3:**
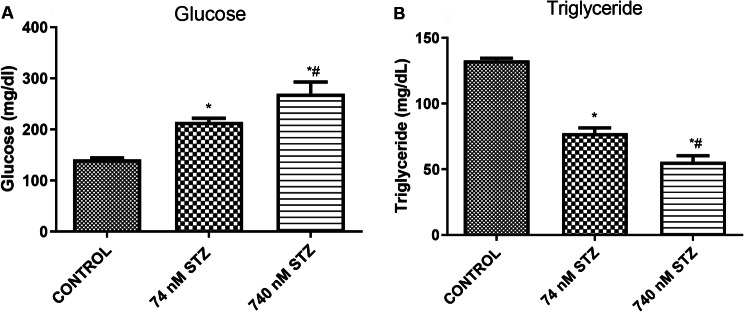
Sugar and lipid levels, 7 days after head STZ injection in cockroaches. *n* = 5. One-way ANOVA with Tukey’s multiple comparisons test indicated a significant increase in (**A**) Glucose levels, and a significant decrease in (**B**) Triglyceride levels in neural tissues of cockroaches exposed to 74 and 740 nM STZ head injection, compared with cockroaches in the control group. All values are mean ± SD. * indicates a significant difference from control; # indicates significant differences from 74 nM STZ injection

**Fig. 4:**
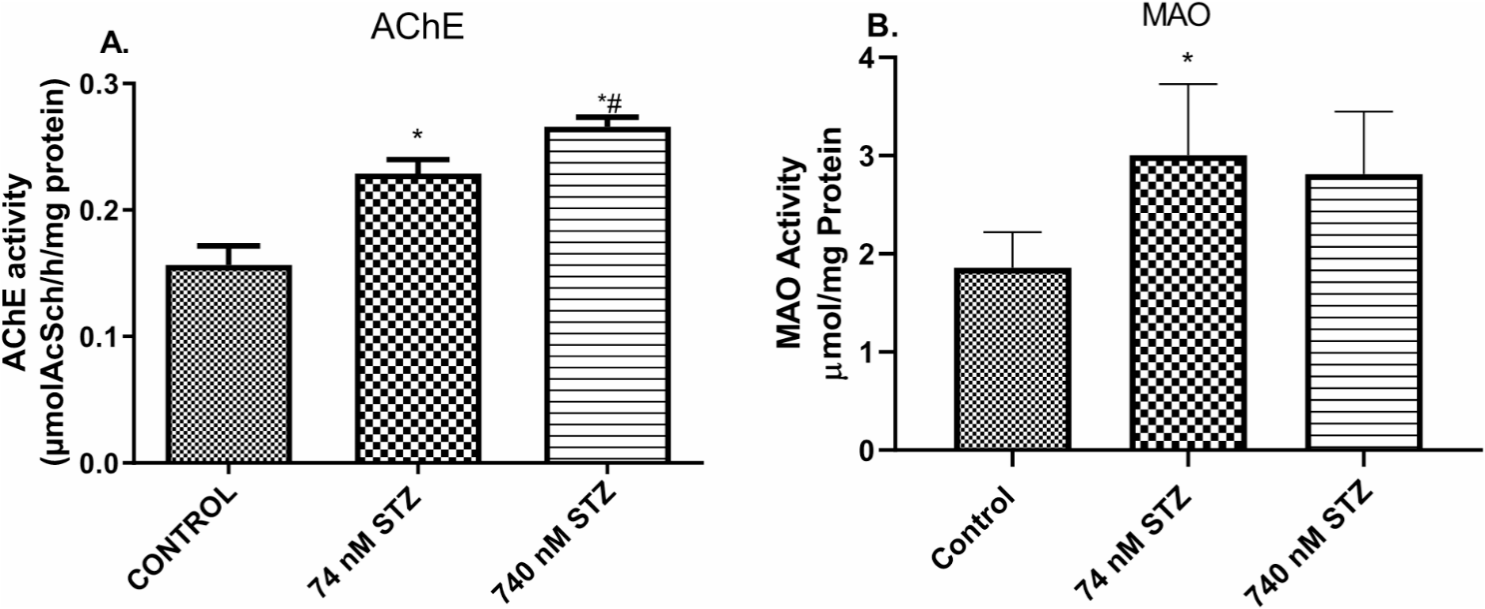
Increased neurotransmitter regulator activity, 7 days after head STZ injection in cockroaches. *n* = 5. One-way ANOVA with Tukey’s multiple comparisons test indicated a significant increase in (**A**) AChE activity and (**B**) MAO levels in in neural tissues of cockroaches exposed to 74 and 740 nM STZ head injection, compared with cockroaches in the control group. All values are mean ± SD. * indicates a significant difference from control; # indicates significant differences from 74 nM STZ injection

Head glucose levels were significantly increased [F (2,9), = 83.77; *P* < 0.0001; Fig. 3A], while triglyceride levels were significantly reduced [F (2,9), = 483.0; *P* < 0.0001; Fig. 3B], and acetylcholinesterase (AChE) [F (2,9), = 91.89; *P* < 0.0001; Fig. 4A] was significantly increased in the 74 and 740 nmol STZ-treated groups compared with control groups. 74 nmol STZ treatment was also significantly different from 740 nmol STZ treatment in the glucose, triglyceride and acetylcholinesterase assays. Monoamine oxidase (MAO) was significantly increased [F (2,9), = 91.89; *P* < 0.0001; Fig. 4B] only in the 74 nmol STZ treated cockroaches compared with control.

**Fig. 5:**
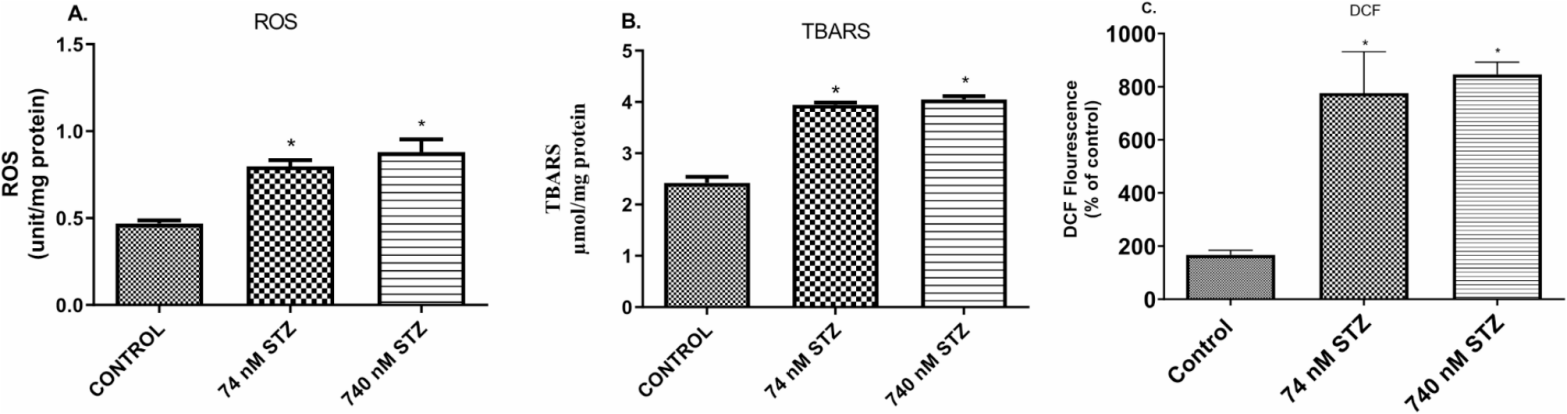
Increased oxidative stress, 7 days after head STZ injection in cockroaches. *n* = 5. One-way ANOVA with Tukey’s multiple comparisons test indicated a significant increase in (**A**) ROS (**B**) TBARS, and (**C**) DCF levels in neural tissues of cockroaches exposed to 74 and 740 nM STZ head injection, compared with cockroaches in the control group. All values are mean ± SD. * indicates a significant difference from control

**Fig. 6:**
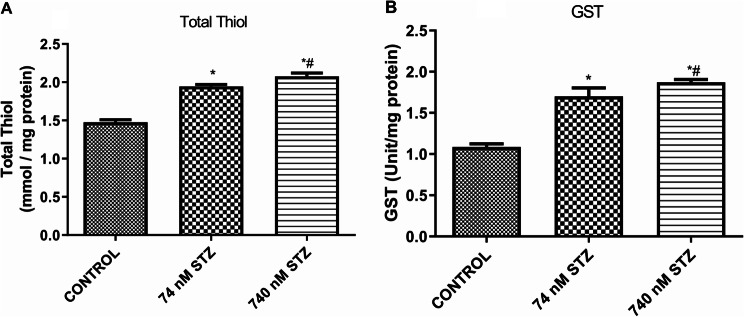
Increased antioxidant and detoxification activity, 7 days after head STZ injection in cockroaches. *n* = 5. One-way ANOVA with Tukey’s multiple comparisons test indicated a significant increase in (**A**) Total Thiol, and (**B**) GST activity in neural tissues of cockroaches exposed to 74 and 740 nM STZ head injection, compared with cockroaches in the control group. All values are mean ± SD. * indicates a significant difference from control. # indicates significant differences from 74 nM STZ injection

Furthermore, we examined the activity of oxidative stress markers in neural tissues and found significantly increased levels of reactive oxygen species (ROS) [F (2,9), = 80.65; *P* < 0.0001; Fig. 5A], malondialdehyde (MDA) [F (2,9), = 514.5; *P* < 0.0001; Fig. 5B], and 2’,7’-dichlorofluorescein (DCF) [F (2,8), = 35.08; *P* = 0.0001; Fig. 5C] in the 74 and 740 nmol STZ treated groups compared with the control group. Antioxidant and detoxification responses were also examined, and we found significantly increased levels of total thiol [F (2,9), = 344.0; *P* < 0.0001; Fig. 6A], and significantly increased activity of Glutathione S-transferase (GST) [F (2,10), = 194.1; *P* < 0.0001, Fig. 6B] in the neural tissues of both 74 and 740 nmol STZ treated cockroaches compared with control. 74 nmol STZ treatment was also significantly different from 740 nmol STZ treatment.

### Gene expression analyses

**Fig. 7:**
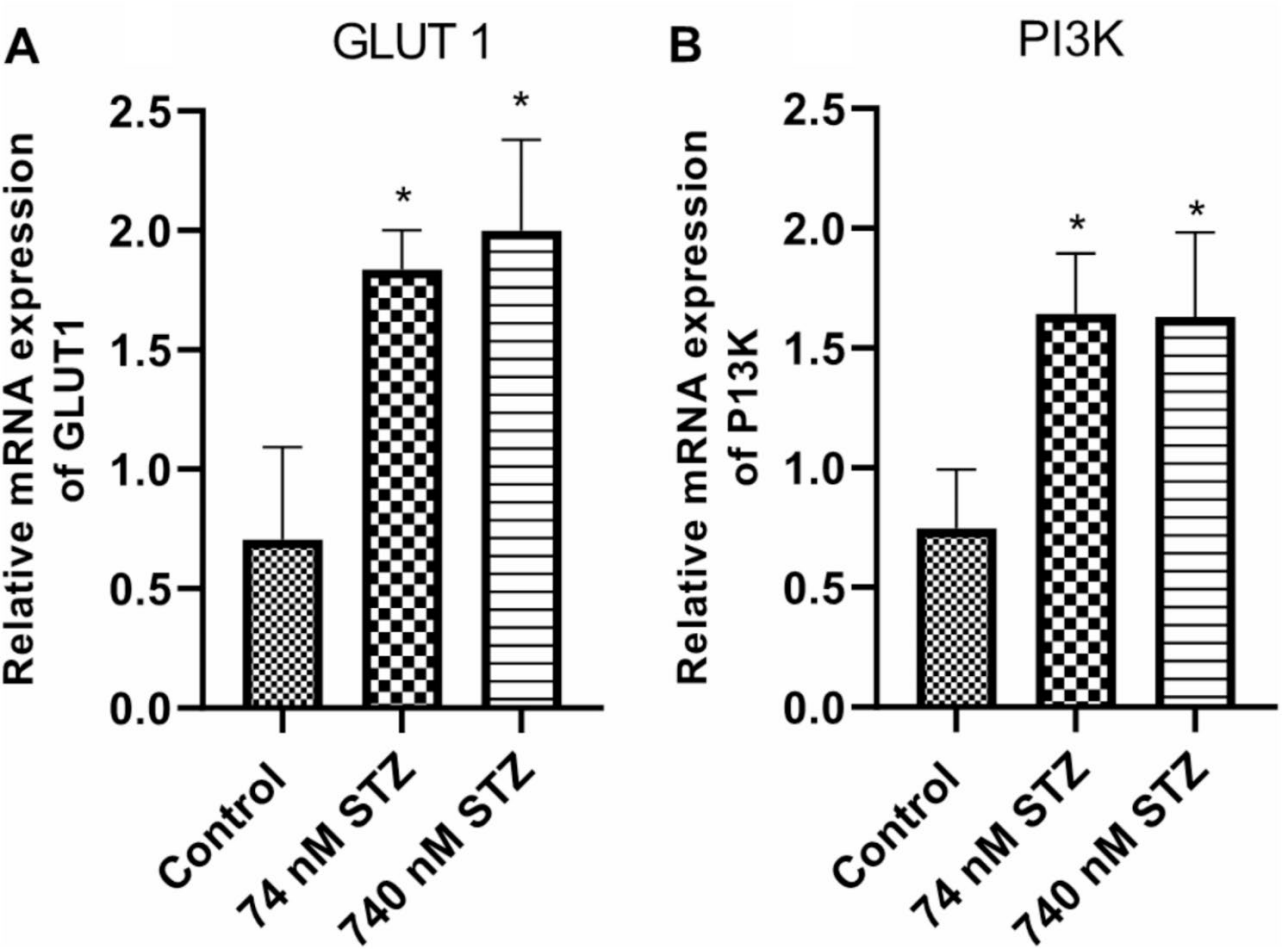
Increased glucose transporter (GLUT 1) activity and phosphoinositide 3-kinase (PI3K) signalling cascade, 7 days after head STZ injection in cockroaches. *n* = 9. One-way ANOVA with Tukey’s multiple comparisons test indicated a significant increase in (**A**) GLUT 1, and (**B**) PI3K activity in neural tissues of cockroaches exposed to 74 and 740 nM STZ head injection, compared with cockroaches in the control group. All values are mean ± SD. * indicates a significant difference from control

**Fig. 8:**
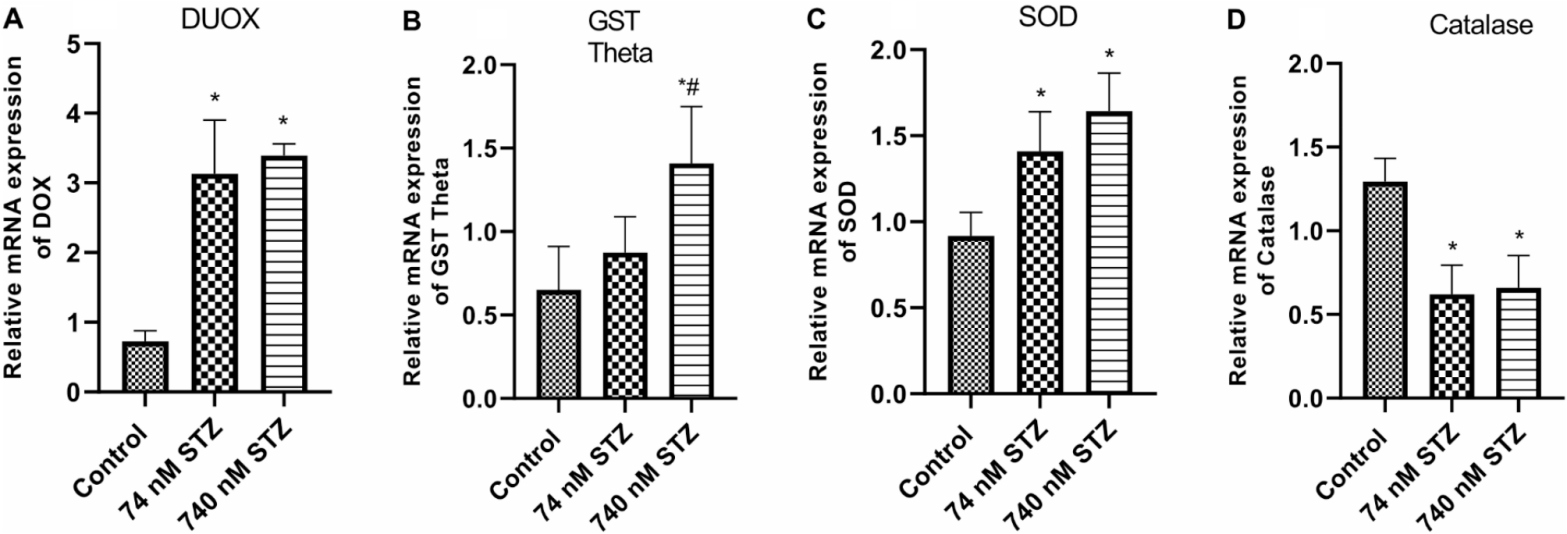
Modulated expression of ROS generation regulator —Dual oxidases (DUOX) and antioxidant/detoxification genes, 7 days after head STZ injection in cockroaches. *n* = 9. One-way ANOVA with Tukey’s multiple comparisons test indicated a significant increase in (**A**) DUOX, (**B**) GST Theta, and (**C**) SOD, as well as significant decrease in (**D**) Catalase in neural tissues of cockroaches exposed to 74 and 740 nM STZ head injection, compared with cockroaches in the control group. All values are mean ± SD. * indicates a significant difference from control; # indicates significant differences from 74 nM STZ injection

**Fig. 9:**
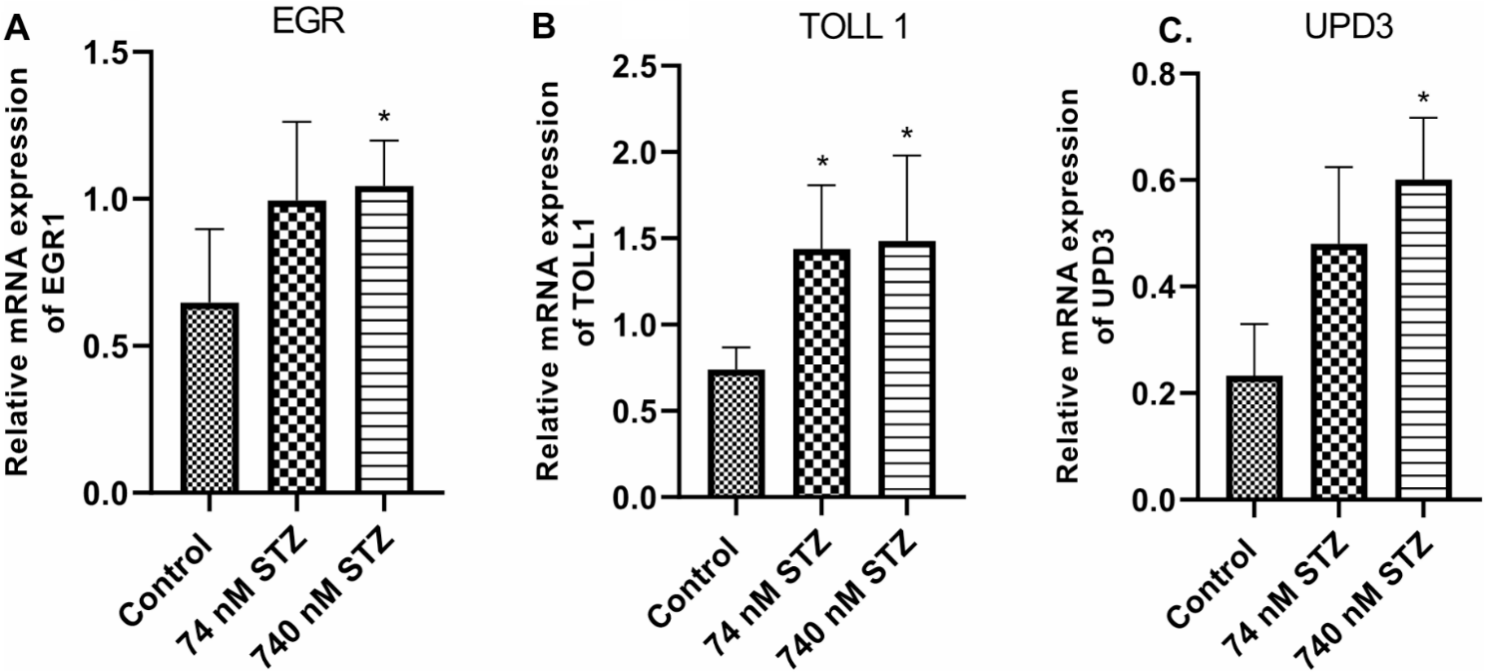
Increased expression of inflammation-associated genes, 7 days after head STZ injection in cockroaches. *n* = 9. One-way ANOVA with Tukey’s multiple comparisons test indicated a significant increase in (**A**) EGR (**B**) TOLL 1, and (**C**) UPD3 activity in neural tissues of cockroaches exposed to 74 and 740 nM STZ head injection, compared with cockroaches in the control group. All values are mean ± SD. * indicates a significant difference from control

We found significantly increased mRNA levels of glucose transporter (GLUT 1) [F (2,14), = 25.20; *P* < 0.0001; Fig. 7A] and phosphoinositide 3-kinase (PI3K) [F (2,14), = 16.54; *P* = 0.0002; Fig. 7B] in the treated groups (74 and 740 nM STZ) compared with control. In the same vein, the mRNA levels of the regulator of ROS generation - Dual oxidase (DUOX) significantly increased in the treated groups compared with the control group [F (2,12), = 50.58; *P* < 0.0001; Fig. 8A], but only the 74 nmol STZ group showed significant increase in the mRNA levels of the detoxification molecule – glutathione S-transferase theta (GST theta) [F (2,14), = 25.20; *P* < 0.0001; Fig. 8B] compared with control. The antioxidant – superoxide dismutase (SOD) [F (2,18), = 20.70; *P* < 0.0001; Fig. 8C] was significantly increased in the 74 and 740 STZ treated groups compared with the control group. Conversely, catalase mRNA levels were significantly reduced in the 74 and 740 nmol STZ treated groups compared with the control group [F (2,13), = 28.54; *P* < 0.0001; Fig. 8D]. Finally, 74 nM head STZ injection induced a significant increase in the mRNA levels of the activators and target genes of the *JNK* pathway - Early growth response protein 1 [EGR: F (2,16), = 5.075; *P* = 0.0196; Fig. 9A], the TOLL/NF-kB pathway – TOLL 1 [F (2,12), = 6.541; *P* = 0.0120; Fig. 9B], and the UPD3/JAK/STAT pathway - unpaired 3 [UPD3: F (2,10), = 9.345; *P* = 0.0051; Fig. 9C] compared with control.

## Discussion

Insects like *Drosophila melanogaster* have been exploited to develop insights into several human diseases [45], and *Nauphoeta cinerea* has been used to understand several mechanistic responses to neurotoxicant exposure [22, 24, 25, 46, 47]. Here, we use N. cinerea – an emerging model of neurotoxicity – to explore neurodegenerative mechanisms, in line with the 3Rs principle to replace, reduce and refine animal models of biomedical research.

Insect survival is rooted in the ability to explore surroundings in the quest for nutrition, mate and escape from predators [48], but certain xenobiotics interfere with insect eclosion and neuromotor competence [18, 21, 25, 49, 50]. For example, organophosphate and pyrethroids inhibit neural acetylcholine breakdown and prolong sodium channel opening respectively, resulting in repeated nerve firing, paralysis and death in insects [51, 52]. An in-depth understanding of insect neurotransmitter regulation may then be used to model mammalian neurodegenerative mechanisms. Indeed, dementia is often modelled with intracerebroventricular (ICV) streptozotocin exposure in rats, which impedes brain glucose metabolism and cholinergic transmission [53]. Streptozotocin exposure reduced *N. cinerea* survival and explorative abilities, similar to the cognitive and motor deficits that are mapped in ICV STZ treated rodents [54].

Despite the brain’s critical need for glucose, excessive GLUT 1 activation predisposes to glucose toxicity via dose-dependent mechanisms [55, 56], and we found increased expression of GLUT 1, along with increased glucose levels in neural tissues of STZ-treated nymphs. Similarly, increased glucose transporter activity has been recorded in ICV STZ exposed rats, with a consequent suggestion that changes in brain glucose metabolism may predispose to neurodegeneration [57]. Given that PI3K downstream signaling induces neuronal apoptosis via upregulated AKT and downregulated ERK signaling [58], and we found increased mRNA expression of PI3K in head STZ injected cockroaches, it is imperative that we further explore PI3K-related neurotoxic mechanisms in the cockroach. Conversely, though hypertriglyceridemia plays a role in insulin resistance that may predispose to neurotoxicity [59], we found reduced triglyceride levels in neural tissues of STZ head injected cockroaches.

Neurotransmitter regulators are current drug targets for mood and attention deficits in humans, as deficiencies in synaptic and neurotransmitter function have been linked with a spectrum of neurodegenerative diseases [60]. Consequently, acetylcholinesterase inhibitors impact varying AChE functions, including the modulation of oxidative stress, inflammation, cell apoptosis and adhesion, while monoamine oxidase inhibitors reduce the degradation of biogenic amines like dopamine, noradrenaline and serotonin to enhance mood [61, 62]. We recorded increased AChE and MAO activities in head STZ-injected cockroaches. In rodent dementia models, unmitigated AChE release in the synaptic cleft exercabates oxido-inflammatory response and the aggregation of pathological proteins like Aβ peptides [62], while MAO-A and MAO-B polymorphisms have been linked with several neuropsychiatric diseases [63]. Although oxidative stress is widely regarded as central to neurodegenerative processes, there are still questions as to whether oxidative stress induces neural degeneration or is a product of dying neurons, and it is still unclear why some nerves are more vulnerable than others [64]. In this study, head STZ injection increased the expression of the ROS generation regulator – DUOX, with a subsequent increase in levels of oxidative stress markers in neural tissues, including ROS, MDA and DCF. Marked oxidative stress has also been recorded in rat models of cognitive impairment induced by ICV STZ injection [26]. Indeed, the generation of reactive oxygen and nitrogen species (RONS) from biological processes or exposure to neurotoxicants predispose to the peroxidation of lipid membranes and impaired proteolysis, which may result in protein aggregation and neurodegeneration [65].

Animal studies have shown promise for antioxidant therapies against neurotoxicity, and we have previously shown *N. cinerea’s* innate ability to increase antioxidant and detoxification systems when exposed to xenobiotics [18, 21, 22, 66], which has been replicated here, as we found increased total thiol levels, GST activity and mRNA levels of GST theta and SOD in neural tissues of head STZ-injected cockroaches, although catalase expression was reduced. Cellular homeostasis attempts to balance RONS generation with antioxidant and detoxification mechanism [65], but this can be overwhelmed by chronic neurotoxicant exposure, and even antioxidant treatments that ameliorate the resulting disease phenotype may not eliminate oxidative stress in rats [67], which may explain why translational efforts, including large randomized controlled trials in humans, have been unable to repeat the success of antioxidant therapies [68]. On the other hand, neuroinflammation plays a role in neurotoxicity [69]. We found increased expression of target genes of the JNK, TOLL and UPD3 inflammation-associated pathways in neural tissues of head STZ-injected cockroaches, like records of proinflammatory cytokine response during neurodegeneration in STZ diabetic rats [70], thereby lending credence to remedies that target the mechanisms of redox-inflammatory crosstalk in neurodegenerative diseases for therapeutic advantage.

## Conclusion and strength of the study

The lobster cockroach can be used to elucidate the mechanisms of chemical-induce neurotoxicity and may potentially serve as a viable model for neurodegeneration, with the possibility of exploiting the cockroach’s neurotransmitter regulators, energy metabolism patterns and redox-inflammatory crosstalk for therapeutic gain. A potentially viable insect model of neurodegeneration also expands the pool of data from which algorithms for new approach methodologies (NAMs) would be built.

### Limitation and future perspectives

The neurodegenerative process is multifactorial, and it presents a plethora of phenotypes that require thorough assessments to ensure accurate characterization. The delineation of neuropathological changes in insect models therefore have to mirror mammalian phenotypes. Hence, immunohistochemical analysis of the cockroach brain is a crucial next step in proving the viability of the model.

## Data Availability

Data used in this study is available upon reasonable request to OBO and OCO.
